# Structural basis of LAIR1 targeting by polymorphic *Plasmodium* RIFINs

**DOI:** 10.1038/s41467-021-24291-6

**Published:** 2021-07-09

**Authors:** Kai Xu, Yiran Wang, Chen-Hsiang Shen, Yiwei Chen, Baoshan Zhang, Kevin Liu, Yaroslav Tsybovsky, Shuishu Wang, S. Katie Farney, Jason Gorman, Tyler Stephens, Raffaello Verardi, Yongping Yang, Tongqing Zhou, Gwo-Yu Chuang, Antonio Lanzavecchia, Luca Piccoli, Peter D. Kwong

**Affiliations:** 1 Vaccine Research Center, National Institute of Allergy and Infectious Diseases, National Institutes of Health, Bethesda, MD USA; 2 Department of Veterinary Biosciences, College of Veterinary Medicine, The Ohio State University, Columbus, OH USA; 3 Institute for Research in Biomedicine, Università della Svizzera italiana, Bellinzona, Switzerland; 4 Institute of Microbiology, ETH Zurich, Wolfgang-Pauli-Strasse 10, Zurich, Switzerland; 5 Electron Microscopy Laboratory, Cancer Research Technology Program, Leidos Biomedical Research, Inc., Frederick National Laboratory for Cancer Research, Frederick, MD USA; 6 Present Address: National Institute of Molecular Genetics (INGM), Milano, Italy

**Keywords:** X-ray crystallography, Malaria

## Abstract

RIFIN, a large family of *Plasmodium* variant surface antigens, plays a crucial role in malaria pathogenesis by mediating immune suppression through activation of inhibitory receptors such as LAIR1, and antibodies with LAIR1 inserts have been identified that bind infected erythrocytes through RIFIN. However, details of RIFIN-mediated LAIR1 recognition and receptor activation have been unclear. Here, we use negative-stain EM to define the architecture of LAIR1-inserted antibodies and determine crystal structures of RIFIN-variable 2 (V2) domain in complex with a LAIR1 domain. These structures reveal the LAIR1-binding region of RIFIN to be hydrophobic and membrane-distal, to exhibit extensive structural diversity, and to interact with RIFIN-V2 in a one-to-one fashion. Through structural and sequence analysis of various LAIR1 constructs, we identify essential elements of RIFIN-binding on LAIR1. Furthermore, a structure-derived LAIR1-binding sequence signature ascertained >20 LAIR1-binding RIFINs, including some from *P. falciparum* field strains and *Plasmodium* species infecting gorillas and chimpanzees.

## Abstract (web summary)

RIFINs are *Plasmodium* surface antigens that suppress the immune response by binding inhibitory receptors such as LAIR1. Here, Xu *et al*. characterize the interaction between RIFIN-variable 2 domain and a LAIR1 domain and identify LAIR1-binding RIFINs in several *Plasmodium* species.

## Introduction

Malaria infects over 200 million people and kills over 400,000 each year^1^. Its causative agents, *Plasmodium* parasites, utilize multiple mechanism to evade the human immune response, including the expression of variable surface antigens on *Plasmodium*-infected erythrocytes (IEs)^2^. With over 150 members, RIFIN comprises one of the larger families of *Plasmodium* variant surface antigens^3,4^ and sequesters parasites by rosetting group A red blood cells, resulting in the development of severe malaria^5^. RIFIN also mediates immune suppression through activation of inhibitory immune receptors including leukocyte-associated immunoglobulin-like receptor 1 (LAIR1) and leukocyte immunoglobulin-like receptor B1 (LILRB1)^6–8^. Notably, human monoclonal antibodies that contain LAIR1-domain insertions in either switch region or between V and DJ regions have been isolated from donors, who have had malaria^8–10^. Some of these antibodies, including MGC34^8^ and MGD21^8^, acquire mutations in the LAIR1 domain during antibody maturation to remove self-reactivity for collagen and to increase affinity for *P. falciparum* isolates through interactions with RIFIN^8^.

To define the structural basis underpinning interactions between wild-type and antibody-inserted LAIR1 and polymorphic RIFIN, we investigated the overall architecture of three types of LAIR1-inserted antibodies, determined crystal structures of LAIR1 in complex with the RIFIN-interactive regions from two RIFINs (PF3D7_1040300 and PF3D7_0401300), and used the resultant structural information to derive a LAIR1-binding signature and to identify RIFINS from diverse *Plasmodium* species capable of recognizing LAIR1.

## Results

### Structural definition of RIFIN-LAIR1 interaction

LAIR1-containing antibodies have distinct insertion modes, including type-1 insertion between V and DJ junctions in CDR H3 region, type-2 insertion in the S region between VH and CH1 region, and type-3 insertion in the S region with deletion of VH and CH1 gene^9^. To gain insight into these three insertion architectures, we determined structures for all three types (Supplementary Fig. 1a–d and Supplementary Table 1). Despite differences in architecture and flexibility, RIFIN binding capacity of LAIR1-insertion appeared similar as assessed by co-IP and electron microscopy (EM) analyses of a chimeric MGJ5/MGD21 antibody in complex with RIFIN (Supplementary Fig. 1e).

To facilitate structural determination of the RIFIN-V2-LAIR1 interaction, we took advantage of the affinity maturation of the LAIR1 ectodomain from antibody MGD21, which increases its affinity to RIFIN (PF3D7_1040300) to 6.2 nM, from lower than 1 µM (Supplementary Fig. 2). Due to its hydrophobicity, RIFIN V2 expresses poorly and has low solubility. We therefore designed a strategy of co-expression and co-purification to obtain the complexes of LAIR1-positive^8^ RIFIN-V2 (PF3D7_1040300, RIF1; and PF3D7_1400600, RIF2) with either LAIR1 ectodomain or MGD21 Fab (Supplementary Fig. 3a, b). The crystal structure of the RIFIN V2 (PF3D7_1040300) in complex with LAIR1 extended to 2.7-Å resolution (Supplementary Table 1), while negative-stain (ns)-EM 3D reconstruction of the MGD21 Fab complex with RIFIN V2 (PF3D7_1400600) indicated the Fab region not to be involved in RIFIN binding (Supplementary Fig. 4).

**Fig. 1:**
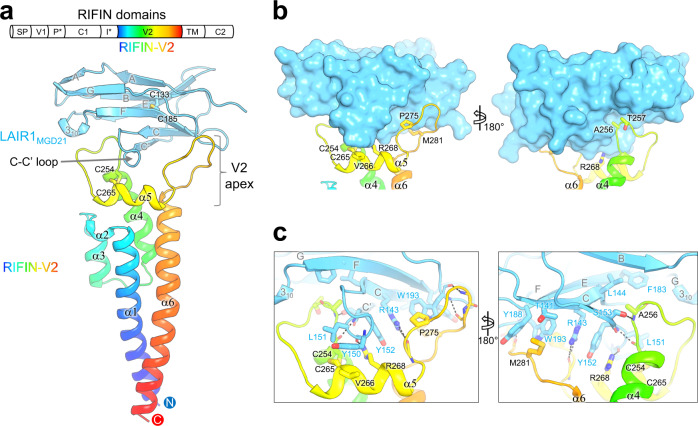
Structure of RIFIN-V2 in complex with LAIR1. **a** Schematic view of A-RIFIN domains (upper, SP: Signal peptide; V1: Variable domain 1; P*: PEXEL motif; C1: Conserved domain 1; I*: Indel motif; V2: Variable domain 2; TM: Transmembrane domain; C2: Conserved domain 2). Structure of RIFIN (PF3D7_1040300) V2 domain in complex with antibody MGD21 LAIR1 domain is presented as cartoon side view (lower). RIFIN-V2 domain is colored in rainbow. LAIR1 domain is colored in cyan. All secondary structural elements are labeled. **b**, Surface and pockets on LAIR-1 that accommodate RIFIN-V2 apex. **c** Detail of RIFIN-LAIR1 interface. Interface residues are shown with side chains as sticks and labeled.

**Fig. 2:**
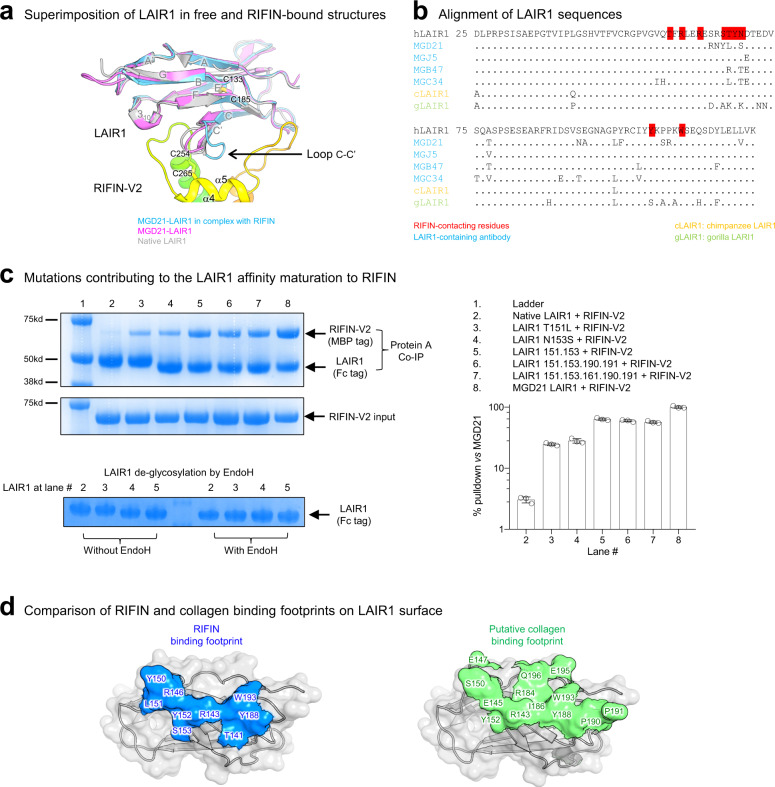
Structural and sequence analysis of various LAIR1 constructs identifies RIFIN-binding essential. **a** Conformational change on LAIR1 c-c’ loop to enable RIFIN binding. **b** Alignment of LAIR1 sequences. (MGD21, hLAIR1: native human LAIR1, MGJ5, MGB47, MGC34, gLAIR1: gorilla LAIR1). **c** Accumulation of critical mutations allows affinity maturation of LAIR1 to RIFIN (upper left panel: SDS-PAGE image; lower left: LAIR1 de-glycosylation by EndoH; upper right panel: SDS-PAGE sample loading list; lower right panel: Quantification of left panel by bar graph. Data are presented as mean values ± SD). *n* = 3 independent measurements. The SDS-PAGE runs were repeated at least twice. **d** RIFIN binding footprint overlapped that of collagen on LAIR1 surface. Note: LAIR1 residue numbering is based on MGD21 construct, which has 84 shift to that of native LAIR1.

The RIFIN-V2-LAIR1 complex resembled a razor with an exchangeable cartridge, where RIFIN V2 is the “handle” and LAIR1 is the “cartridge” (Fig. 1a). The RIFIN-V2 portion of the complex comprised 6 helices (α1-α6) (Supplementary Fig. 5a). Two longer helices, α1 and α6, and two shorter helices, α3 and α4, formed a four-helix bundle with helices α2 and α5 packed perpendicularly to the bundle. Helix α5 and its adjacent loops (α4-α5 and α5-α6) were connected by a disulfide bond (C254-C265) between α4 and α5; this region (hereafter referred to as the apex region) is likely located distally from the membrane, as the carboxyl terminus of the V2 region connects directly to the transmembrane. Comparison to a previously determined structure of RIFIN-V2 (PDB 6ZDX^7^) revealed only ~10% sequence identity and extensive variation in length of helices (Supplementary Fig. 5c,d). The LAIR1 ectodomain portion of the complex comprised two β sheets along the lines of a standard V-type immunoglobulin fold^11^, with a disulfide bond (C133-C185) connecting strands B and F and linking the two β sheets (Fig. 1a). Comparison to the previously determined structure of ligand-free LAIR1 (PDB 3KGR^11^) suggested the binding of RIFIN to induce conformational alterations in the C-C’ loop (Fig. 2a), although their overall structures were essentially identical with a root-mean-square deviation (rmsd) of 0.41 Å over all Cα atoms. Interestingly, the interface between RIFIN-V2 and LAIR1 buried a surface area of 917 Å^2^ on each protein. The interface consisted of backbone-mediated hydrogen bonds and extensive hydrophobic interactions with highly complementary shapes matching between RIFIN and LAIR1. The RIFIN-V2 apex clamps the side of LAIR1, interacting with loop C-C’, strand C, and the loop neighboring strand F. Residues on RIFIN involved in binding LAIR1 included C254, S255, A256, T257 on loop α4-α5, C265, V266, R268 on α5, and P275, M281 on loop α5-α6 (Fig. 1b, c). Interface residues on the LAIR1 included T141, R143 on strand C, R146, Y150, L151 on loop C-C’, Y152, S153 on strand C’, Y188, W193 in the loop next to strand F. Hydrophobic stacking was observed between LAIR1 Y150, L151 and RIFIN I261, C254-C265 disulfide bond. Planar side chain stacking was observed between Y188, W193, R143, Y152 of LAIR1, and R268 and C254-C265 disulfide bond of RIFIN (Fig. 1c, and Supplementary Fig. 5f).

The revealed mode of RIFIN interaction with a lateral side of the LAIR1-immunoglobulin domain is strikingly different from the recently determined structure of RIFIN-V2 (PF3D7_1254800) in complex with the inhibitory receptor, LILRB1^7^ (Supplementary Fig. 5c-d). Collectively, these results provide atomic-level details of RIFIN interaction with LAIR1 and exemplify both the remarkable diversity of RIFINs as well as the distinct modes of RIFIN interaction with cellular factors.

### Structure-function analysis of LAIR1-RIFIN interaction

To gain insight into the role of LAIR1 affinity maturation in LAIR1-RIFIN interaction, we designed a series of LAIR1 constructs with accumulating mutations in the RIFIN-binding interface, starting from unmutated (native) LAIR1 and extending to the affinity-matured LAIR1 in MGD21, and assessed their binding to RIFIN by co-immunoprecipitation (Fig. 2b, c). Native LAIR1 has an *N*-linked glycosylation sequon at residue N153, which can be de-glycosylated by Endoglycosidase H (EndoH) treatment (compare the position of LAIR1 in lanes 2 and 3 with that in other lanes in Fig. 2c upper and lower left panel). We found an N153S de-glycosylating mutation at the binding interface to enhance RIFIN binding. Mutation T151L hydrophobically interacted with C254-C265 in RIFIN V2 (Fig. 1c). Both N153S and T151L LAIR1 mutations substantially increased affinity to RIFIN. By contrast, affinity maturation changes at positions 161, 190, and 191, which are only involved in collagen binding^11^, had no detectable impact on RIFIN binding (Fig. 2c).

The binding footprints of collagen and RIFIN on LAIR1 were distinct but overlapping (Fig. 2d). Several putative critical LAIR1 residues involved in collagen binding, such as P190 and P191, were mutated in LAIR1-inserted antibodies including MGD21, thus reducing the collagen self-reactivity of these antibodies. Overall, these structure-function studies show how B cells expressing antibodies with inserted LAIR1 can reduce collagen affinity without impacting LAIR1 affinity and how native LAIR1 can acquire a ~10-fold increase in affinity to RIFIN through single amino acid substitutions at either position 151 or 153; this affinity increase provides biological context for the affinity matured LAIR1 used in our structural definition of the LAIR1-RIFIN interaction as well as insight into the selection and maturation of LAIR1-containing antibodies. Comparing with the affinity matured LAIR1 on the antibodies, the native LAIR1 receptor had a much-reduced affinity to the RIFIN tested in this study, in a similar range to the recently reported RIFIN-LILRB1 interaction^7^. This possibly reflects an optimal balance of parasitic functions between immune receptor activation and avoidance of immune cell attachment.

### Delineation of LAIR1-binding sequence signature in RIFIN

**Fig. 3:**
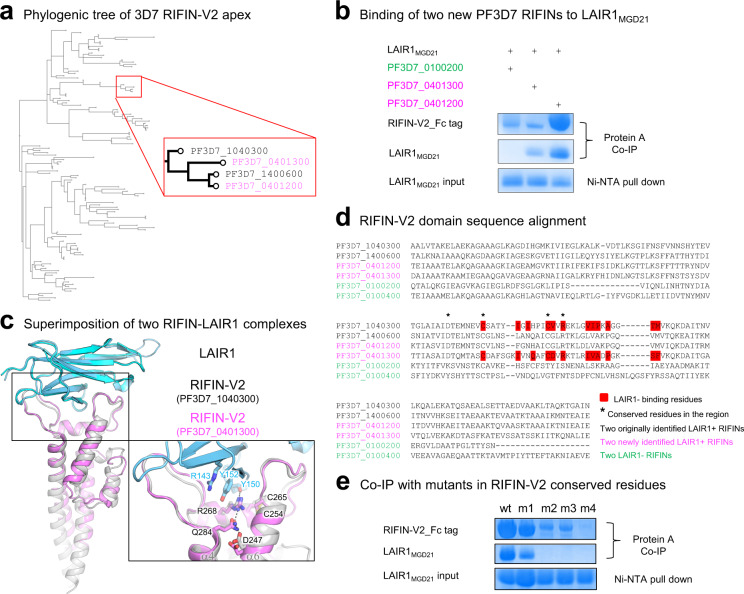
LAIR1 targets a conserved interface within a subset of RIFIN family members. **a** Phylogenic analysis of *plasmodium* strain 3D7 RIFIN-v2 apex identified two additional RIFINs in the same branch as known LAIR1-binding RIFINs. **b** Co-IP assay confirmed the MGD21.LAIR1-binding by the two new RIFINs. The SDS-PAGE runs were repeated at least twice. **c** Superimposition of two RIFIN-LAIR1 complex structures. **d** Sequence comparison of MGD21-positive (cyan and magenta) and MGD21-negative (green) RIFINs V2 region, with LAIR1-binding region highlighted in red and conserved residues near the binding region marked with asterisks (*). **e** Co-IP with Alanine mutants in RIFIN-V2 four conserved residues. wt: wildtype PF3D7_1040300, m1: D247A, m2: C254A, m3: C265A, m4: R268A. The SDS-PAGE runs were repeated at least twice.

Sequence conservation analysis of 158 PF3D7 A-type RIFIN sequences indicated that, although the C254-C265 disulfide bond in the V2 domain is conserved, residues corresponding to the LAIR1-binding region were highly variable (Supplementary Fig. 5c). Phylogenetic analysis of the RIFIN-V2 apex sequence revealed two additional 3D7 RIFINs, referred to as RIF3 (PF3D7_0401300) and RIF4 (PF3D7_0401200), to be in the same branch as the initially identified LAIR1-positive RIFINs (RIF1 and RIF2) (Fig. 3a). A similar approach also identified seven RIFINs in non-3D7 strains. Co-immunoprecipitation (co-IP) assays confirmed LAIR1 interaction with the newly identified 3D7 RIFINs, RIF3 and RIF4 (Fig. 3b), and with three of the non-3D7 RIFINs (Supplementary Fig. 6a, b).

To provide insight into the recognition of these RIFINs, we determined the structure of the RIF3-V2 domain in complex with MGD21 LAIR1 at 2.5 Å resolution (Table 1). The RIF3-LAIR1 structure resembled that of RIF1-LAIR1 with an overall rmsd of 1.46 Å over 546 Cα atoms (Fig. 3c), despite clear diversity in the primary RIFIN sequences at the binding interface (Fig. 3d). Structural analysis and sequence comparison of the LAIR1-RIFIN complex suggested a set of four conserved residues contributing to LAIR1-binding capacity, including residues D247, C254, C265 and R268. D247 forms hydrogen bonds with Q284 to stabilize interaction between α4 and α6; C254-C265 forms a disulfide bond connecting α4 and α5; and R268 not only connects α5-α6 by hydrogen-bonding with Q284, but also forms multiple interactions with LAIR1 by π-stacking with Y152_LAIR1_ and by hydrogen bonding with the main chain carbonyl of Y150_LAIR1_ (Figs. 3c and 2d). We further verified the contribution of the four conserved RIFIN residues to the LAIR1-binding capacity by co-IP assay with alanine mutations. The D247A mutation significantly reduced affinity between RIFIN and LAIR1, and all other single mutations completely knocked out the LAIR1 binding capacity of RIFIN (Fig. 3e).

**Table 1.** Statistics of X-ray diffraction data collection and structural refinement.

	LAIR1-RIFIN.V2 PF3D7_1040300 (RIF1) (NH_4_)_2_PtCl_4_	LAIR1-RIFIN.V2 PF3D7_0401300 (RIF3)	MGC34 Fab	MGD21 Fab
PDB accession code	7JZI	7JZK	7JZ1	7JZ4
**Data collection**				
Wavelength	1.072	1	1	1
Resolution range (Å)	50-2.70 (2.75-2.70)	50-2.45 (2.49-2.45)	50-3.35 (3.47-3.35)	50-2.75 (2.80-2.75)
Space group	C 2	C 2	P 6_5_ 2 2	P 2_1_
Cell dimensions				
*a, b, c* (Å)	144.4, 72.2, 57.9	87.1, 95.8, 71.8	101.4, 101.4, 319.9	80.9, 81.1, 111.6
α, β, γ (°)	90, 91.1, 90	90, 104, 90	90, 90, 120	90, 94, 90
Unique reflections	16105 (1480)	19253 (1854)	14543 (1399)	37566 (3513)
Multiplicity	7.2 (5.6)	3.0 (2.7)	4.3 (3.7)	3.8 (3.8)
Completeness (%)	98.8 (94.6)	92.3 (93.9)	99.7 (100.0)	99.9 (99.5)
I / σI	42.7 (1.8)	14.1 (2.0)	9.2 (1.1)	16.1 (1.8)
Wilson *B*-factor	88.80	43.17	110.38	69.04
*R*_merge_	0.070 (0.593)	0.104 (0.423)	0.177 (0.784)	0.103 (0.872)
CC_1/2_	0.900	0.855	0.603	0.641
**Refinement**				
Reflections used in refinement	16092 (1479)	19219 (1847)	14536 (1399)	37552 (3511)
Reflections used for R-free	1612 (152)	914 (81)	687 (62)	1844 (172)
*R*_work_	0.22 (0.38)	0.21 (0.27)	0.24 (0.32)	0.20 (0.31)
*R*_free_	0.26 (0.40)	0.26 (0.33)	0.26 (0.29)	0.25 (0.35)
Number of non-H atoms	3430	3468	4276	8516
macromolecules	3417	3371	4276	8453
ligands	2	39		53
solvent	11	58		10
Protein residues	450	442	564	1109
RMS(bonds) (Å)	0.008	0.010	0.005	0.005
RMS(angles) (°)	1.41	1.47	0.97	0.91
Ramachandran favored (%)	98. 41	99.54	93.39	95.43
Ramachandran outliers (%)	0.00	0.23	0.00	0.00
Average *B*-factor (Å^2^)	111.11	54.57	107.84	82.22
macromolecules	111.03	53.86	107.84	81.79
ligands	276.68	117.73		154.29
solvent	106.01	53.32		60.89

Values in parentheses are for highest-resolution shell.

**Fig. 4:**
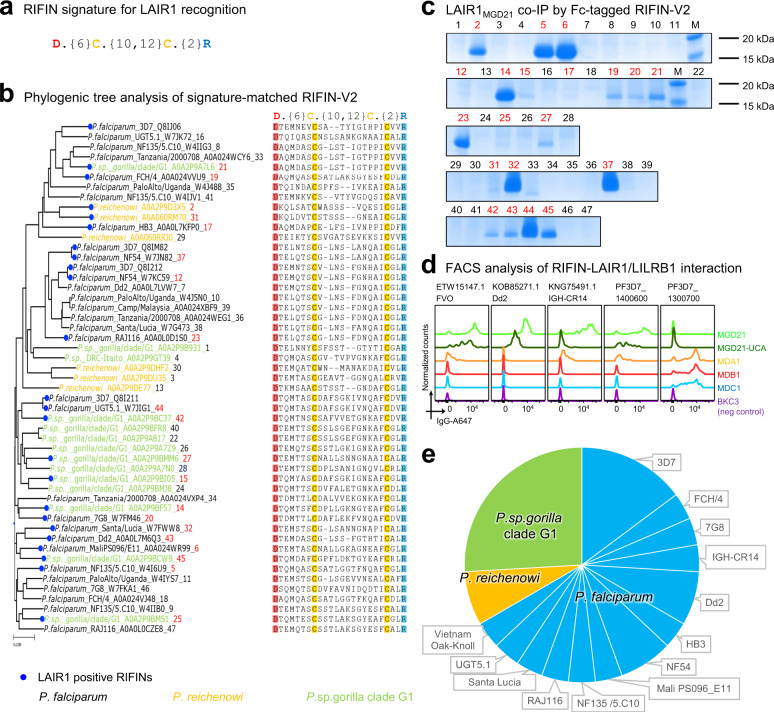
Signature-based search identifies LAIR1-binding RIFINs from *Plasmodium* sequences. **a** Structure-based delineation of LAIR1 binding signature on RIFIN. **b** Phylogenic tree of V2 domain showing name and apex sequences of signature-matched RIFINs. Blue circles indicate the empirically confirmed LAIR1-positive RIFIN. Ape-infecting strains are highlighted in yellow or green, and *P*. *falciparum* strains in black. The number at the end of each name indicates its sequence in (**c**). **c** Coomassie blue-stained SDS-PAGE showing LAIR1_MGD21_ co-immunoprecipitation (Co-IP) by Fc-tagged RIFIN-V2 constructs identified by the signature. Confirmed LAIR1-binding RIFIN ID are highlighted in red. Lanes marked with “M” are molecular ladders showing 15–20 kDa bands. The SDS-PAGE runs were repeated at least twice. **d** FACS analysis example of RIFIN-LAIR1 (MGD21 and MGD21-UCA) or RIFIN-LILRB1(MDA1, MDB1, and MDC1) interaction. **e** Pie chart summary of 27 experimentally confirmed LAIR1-positive RIFINs, colored by three *Plasmodium* species (*P. falciparum* and two ape-specific species).

To facilitate the identification of additional LAIR1-binding RIFIN, we incorporated the above four conserved residues in a LAIR1 recognition signature (Fig. 4a), and further identified 47 signature-matched RIFINs from the *Plasmodium* genome database (Fig. 4b). By comparison, a “three-residue” signature without D247 identified 92 signature-matched RIFINs (Supplementary Data 1). Co-immunoprecipitation confirmed LAIR1 binding for 20 of the 47 RIFINs with the four-residue signature (Fig. 4c and Supplementary Fig. 7a). A fluorescence-activated cell sorting (FACS) assay further showed that the surface of Expi293F cells expressing full-length constructs of four selected RIFIN whose binding to LAIR1 was confirmed by immunoprecipitation to be stained by both MGD21 antibody and native LAIR1, but not by LILRB1-inserted antibodies, MDA1, MDB1, and MDC1^12^ or by a negative control antibody (Fig. 4d and Supplementary Fig. 7b). Taxonomy analysis indicated the 27 RIFINs with confirmed LAIR1 binding to be from multiple *P. falciparum* field strains or non-human primate-specific *Plasmodium* species, including *P. sp. gorilla* clade G1, which infects gorillas, and *Plasmodium reichenowi*, which infects chimpanzees (Fig. 4e).

## Discussion

Only a small subset of RIFIN family members can interact with LAIR1^6^; however, the structural and bioinformatic analyses performed here allowed us to delineate a sequence signature for LAIR1-interacting RIFINs, by delineating critical residues at the RIFIN apex and their contribution to LAIR1 specificity. Binding to LAIR1 was confirmed in 20 of the 47 signature-matched RIFINs. These results demonstrate LAIR1-binding RIFINs to exist in multiple, evolutionarily-related, *Plasmodium* species capable of infecting humans, chimpanzees and gorillas, suggesting a link in the evolutionary origin of the human malaria parasite^13^.

The “razor and cartridge” mode of interaction between RIFIN and LAIR1 that we define here may constitute a general structural mechanism used by RIFIN to target inhibitory receptors^12^. The interface between RIFIN-V2 apex and LAIR1 C-C’ region largely comprises hydrophobic interactions and backbone-mediated hydrogen bonds, and the interface is facilitated by an induced conformational fit of flexible RIFIN-V2 apex loops. These structural and chemical properties appear to allow for promiscuous interactions between the LAIR1 receptor and diverse *Plasmodium* RIFINs, suggesting RIFIN-mediated LAIR1-targeting may be a conserved strategy allowing for immune suppression across multiple *Plasmodium* species of malignant malaria, possibly inherited from common ancestors.

In terms of immune recognition of LAIR1-targeting RIFINs, we note that the antigenic diversity of RIFINs^3,4,14^, the limited surface coincidence of different *rifin* genes^5^, and the rapid switching of variant surface antigens^15^ may allow for effective evasion of host immune targeting. Collectively, the presence of multiple LAIR1-binding RIFINs and their polymorphism provides evidence for the importance of targeting inhibitory receptors as well as a means to escape immune responses targeting specific RIFINs. The extensive sequence diversity of the LAIR1-binding surface of RIFIN—even among those RIFINS that we demonstrated to be positive for LAIR1 binding by co-immunoprecipitation—along with the sequence conservation of LAIR1s from hosts infected by malaria (Fig. 2b) highlights the complex polymorphic interactions that malaria parasites make with host factors to enhance their survival. In this context, we note that receptor-based antibodies, such as the LAIR1-inserted antibodies analyzed here, have been optimized by B cell-based mechanisms of affinity maturation (Fig. 2c) and may thus exemplify “less-escapable” solutions for pathogen recognition.

## Methods

### Construct and protein preparation

LAIR1 (wild type and mutants), antibodies (MGC34, MGD21, MGJ5, MGM1) and RIFIN genes were synthesized and subcloned (GenScript, NJ) into pVRC8400 vectors, with HRV3C cleavable His or Fc tag. DNAs were transfected into either Expi293F cells or Expi293 GnTI- cells (Thermo Fisher) using Turbo293 transfection reagent (Speed BioSystems). Six days post transfection, culture supernatants were harvested, and affinity purified with cOmplete™ His-Tag Purification Resin (Roche) or protein A resin (GE) by following manufacture’s protocols.

### Affinity measurement

Affinity of native LAIR1 and MGD21 to RIFIN_V2 domain was assessed using a fortéBio Octet Red384 instrument. His-tagged RIFIN V2 domain was immobilized on Ni-NTA biosensors, then dipped into either LAIR1 or MGD21 in the twofold concentration series. Sensorgrams of the concentration series were corrected with corresponding blank curves and fitted globally with Octet evaluation software using a 1:1 Langmuir model of binding.

### Co-IP assay

Protein complex co-immunoprecipitation were performed by incubating condition media with protein A resin for one hour at room temperature. Fc-tagged RIFIN or LAIR1 were pulled down by protein A resin, while the non Fc-tagged proteins were co-pulled down. The co-IP protein complexes were analyzed with SDS-PAGE and visualized by staining with Coomassie blue. SDS-PAGE gel band intensity was quantified with ImageJ (https://imagej.nih.gov/).

### Negative-staining EM analysis

Samples were diluted with a buffer containing 20 mM HEPES, pH 7.0, 150 mM NaCl, adsorbed to a freshly glow-discharged carbon-film grid, washed with the above buffer, and stained with 0.7% uranyl formate. Images were collected at a magnification of 100,000 using SerialEM^16^ on a FEI Tecnai T20 microscope equipped with a 2k x 2k Eagle CCD camera and operated at 200 kV, or at a magnification of 57,000 using EPU on a ThermoFisher Talos F200C microscope equipped with a 4k x 4k CETA 16 M camera and operated at 200 kV. The pixel size was 2.2 Å for the Eagle CCD camera and 2.5 Å for the CETA camera. Particles were picked using e2boxer from the EMAN2^17^ software package. Reference-free 2D classification was performed using EMAN2 and SPIDER^18^. 3D reconstruction was performed using SPIDER, cryoSPARC^19^ and FREALIGN^20^, with initial 3D references generated with EMAN2.

### Crystal screening and X-ray crystallographic data collection

To facilitate crystallization, proteins and complexes were treated with HRV3C to remove affinity tags and further purified by SEC with Superdex 200 chromatography column (GE) in the HEPES buffer (5 mM HEPES pH7.5 and 150 mM NaCl). Crystallization conditions were screened using Hampton Research, Wizard, and QIAGEN crystal screening kits. Crystal plates were set up using a Mosquito crystallization robot. Crystals initially observed from the wells were manually reproduced. MGC34 crystal grew in 0.05 M CaCl_2_, 0.1 M BIS-TRIS pH 6.5 and 30% v/v PEG MME 550. MGD21 crystal grew in 0.1 M MgCl_2_, 0.1 M Tris HCl pH 8.5, 20% PEG 3350 and 25% PEG 400. RIF1-LAIR1 complex crystal grew in 0.085 M HEPES pH 7.5, 17% PEG 4000, 15% Glycerol and 8.5% Isopropanol. RIF3-LAIR1 complex crystal grew in 0.1 M NaCl, 0.1 M Tris HCl pH 8.5 and 20% PEG 3350. Optimized crystals were cryoprotected in 30% glycerol and flash-frozen in liquid nitrogen. Platinum derivatives of RIF1-LAIR1 complex crystal were prepared by soaking the crystal in well solution supplemented with various concentration of (NH_4_)_2_PtCl_4_ for 1 h. Data were collected at a wavelength of 1.00 Å, except Platinum derivative (1.072 Å), at the SER-CAT beamline ID-22 (Advanced Photon Source, Argonne National Laboratory).

### Structural determination

Diffraction data were processed with the HKL2000 suite^21^. Phasing solution of RIF1-LAIR1 structure were determined by MR-SAD in Phenix^22^ with combination of molecular replacement (LAIR1 template: 3KGR) and Platinum signal. Other structural solution was obtained by molecular replacement with Phaser in Phenix using search models consisting of the variable region generated with PIGSpro^23^ and the constant regions. Model building was carried out with Coot^24^. Refinement was carried out with Phenix. Data collection and refinement statistics are shown in Table 1.

### Bioinformatic analysis

Pairwise RIFIN V2 structural comparison was perform with FATCAT server^25^. RIFIN structural conservation against structures in the Protein Data Bank was analyzed with Dali server^26^. To calculate sequence conservation, all type A RIFIN sequences of strain 3D7 were downloaded from NCBI. Duplicate sequences were removed. The remaining 117 sequences were aligned with clustalW^27^. The Shannon entropy was calculated for each residue, yielding a minimum value of 0.282 and a maximum value of 2.352. Shannon entropy values were then assigned to the B-factor column of the PDB file and the structure was colored by Shannon entropy using PyMOL. The signature of LAIR1 binding RIFIN was defined by the conserved amino acids that contribute to the interface and the structural integrity of V2 apex. The complete protein sequences of Malaria RIFIN were downloaded at Nov-06-2019 from UniProt, from which the V2 domain sequences were extracted for signature analysis. Phylogenetic analysis was first performed by aligning the V2 domain sequences with Mafft^28^, then a neighbor joining tree was constructed by using clustalW^27^. Python library ETE^29^ was used to visualize phylogenetic tree.

### FACS analysis

To validate whether selected RIFINs are the antigens for LAIR1-containing antibodies MGD21 or germ-line reverted MGD21-UCA, gene encoding V2 region of RIFIN candidates were produced by gene synthesis (Genescript) and cloned into pDisplay vector (Invitrogen) that contains a hemagglutinin (HA) tag. RIFIN-containing pDisplay vectors were transiently transfected into Expi293F cells respectively (ThermoFisher Scientific) using PEI. 72 h post-transfection, cells expressing RIFINs were collected and stained with MGD21, MGD21UCA, LILRB1-containing antibodies MDA1, B1, C1^12^ or control IgG antibodies BCK3 and tested by flow cytometry. Briefly, 5 μg/ml of testing antibodies were added to the RIFIN-transfected cells for 30 min at 4 °C and followed by washing steps before staining with secondary of 2.5 μg/ml of Alexa Fluor 647-conjugated goat anti-human IgG antibody (Jackson ImmunoResearch, catalog no. 109-606-170). The cells were washed again, and then stained with 5 μg/ml of rabbit anti-HA tag for the same condition followed by second washing before adding Alexa Fluor 488-conjugated goat anti-rabbit IgG (Life Technologies, catalog no. A11034) for the detection of RIFIN binding. Dead cells were excluded by gating. FACS was performed in BD FACSCanto I (Cat no. 337175). Gating followed the standard gating procedures. Under the pseudocolor plot, live cells were in grouped in clear population in the FSC/SSC plot. The Singlets were gated by eliminating the shades of the edge. RIFIN-HA-488 positive population was determined by comparing to the unstained cells. IgG-A647 was visualized using histogram. FACS Diva (version 6.2) was used for acquisition of samples. Flow-Jo (version 10.1) was used for all the FACS analyses. A figure exemplifying the gating strategy is provided in the Fig S7b.

### Reporting summary

Further information on research design is available in the Nature Research Reporting Summary linked to this article.

## Supplementary information

Supplementary Information

Description of Additional Supplementary Files

Supplementary Data 1

Reporting Summary

## Data Availability

Coordinates for the crystal structures determined in this study have been deposited in the Protein Data Bank as PDB 7JZ1, 7JZ4, 7JZI and 7JZK. PDB 3KGR and 6ZDX were downloaded from Protein Data Bank. These data were analyzed in Figs. 1, 2, 3 and Supplementary Figs. 1, 5. Raw data (SDS-PAGE gel image) associated with Figs. 2c, 3b, 3e, 4c, S6 and S7a are provided in Source data file. *Plasmodium* genome database can be accessed on the web (plasmodb.org). All relevant data are provided with this paper and also available from the authors. Source data are provided with this paper.

## Source data

Source Data
