# Supplementary material for: Structural basis of LAIR1 targeting by polymorphic Plasmodium RIFINs
Source: Nat Commun. 2021 Jul 9;12:4226. doi: 10.1038/s41467-021-24291-6 (PMC8270905; doi:10.1038/s41467-021-24291-6)
Supplement: Supplementary file 1 — Supplementary Information [file 41467_2021_24291_MOESM1_ESM.pdf]

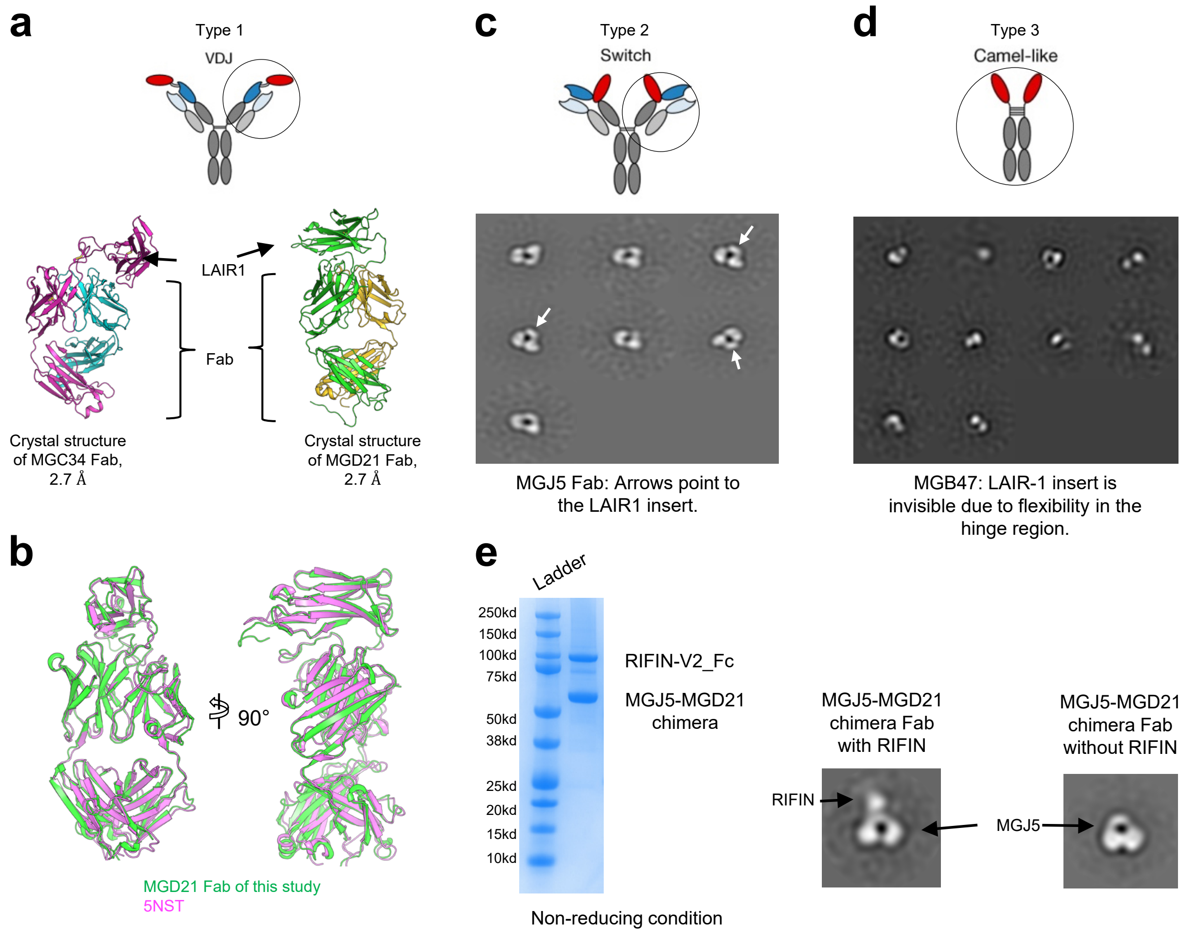

Supplementary Fig. 1 Architectural diversity of LAIR1-inserted antibodies. a, Crystal structure of two type 1 LAIR1-containing antibody Fab domains, including MGC34 and MGD21. **b,** Superimposition of MGD21 Fab structure with PDB ID 5NST. **c,** and **d**, ns-EM images of type 2 (MGJ5) and type 3 (MGB46) LAIR1-containing antibodies. **e**, Recognition of RIFIN by type 2 LAIR1 inserted antibodies MGJ5 and MGM1 chimera with MGD21-LAIR1 swapped in. nsEM image of MGJ5 chimera with or without RIFIN-V2 domain (PF3D7_1040300) (right panel). The SDS-PAGE runs were repeated at least twice.

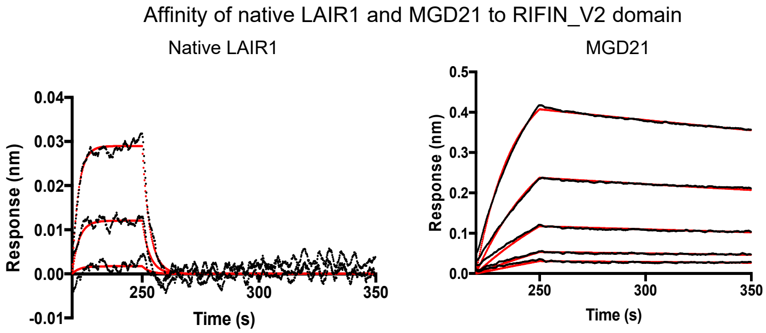

Supplementary Fig. 2 RIFIN-V2 domain mediates LAIR1 binding. Affinity of native and matured LAIR1 to RIFIN (PF3D7_1040300) V2 domain.

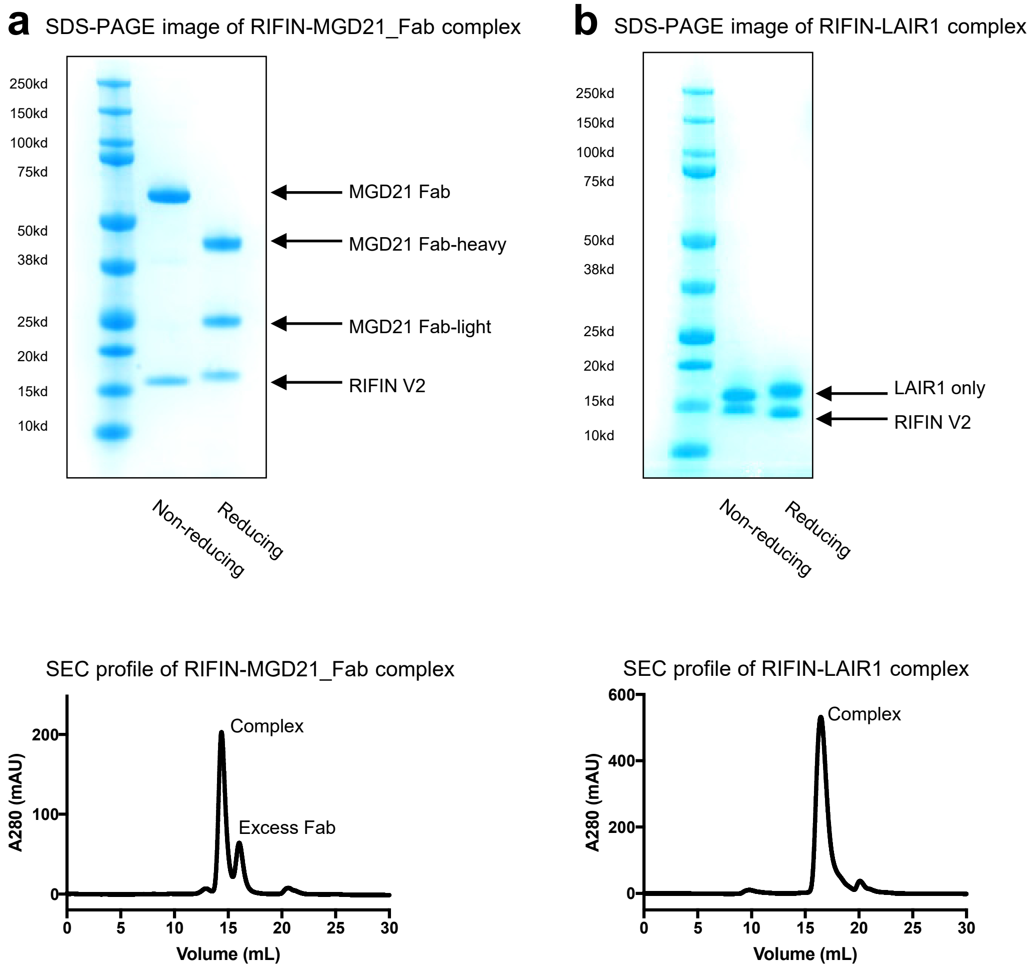

Supplementary Fig. 3 Expression and purification of LAIR1 and RIFIN constructs. a, Upper panel: SDS-PAGE image of RIFIN-MGD21_Fab (PF3D7_1400600) complex. Lower Panel: SEC profile of RIFIN-MGD21_Fab (PF3D7_1400600) complex. The SDS-PAGE runs were repeated at least twice. **b**, Upper panel: SDS-PAGE image of LAIR1-RIFIN (PF3D7_1040300) complex. Lower Panel: SEC profile of LAIR1-RIFIN (PF3D7_1040300) complex. The SDS-PAGE runs were repeated at least twice.

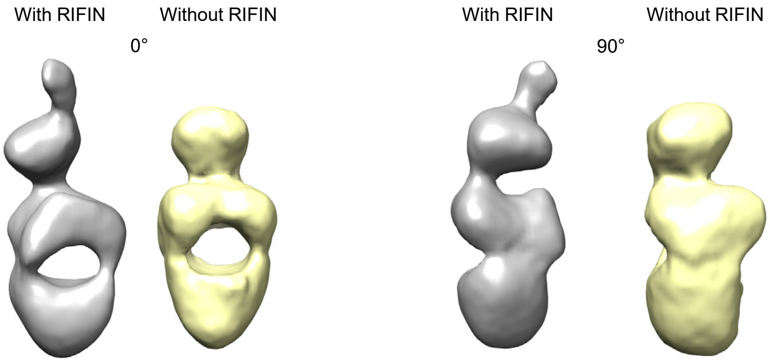

Supplementary Fig. 4 3D reconstruction of nsEM image of MGD21 Fab alone and in complex with RIFIN-V2 (PF3D7_1400600). Maps are superimposed to each other and shown in orthogonal views.

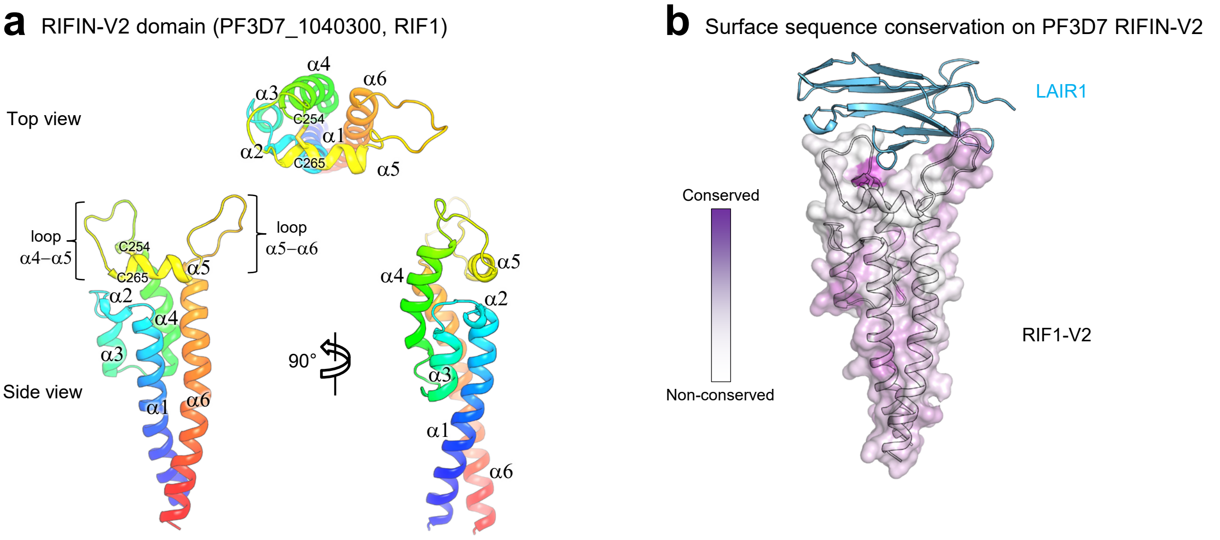

c Pairwise alignment (Identity 11.93% Similarity 25.69%)

```
PF3D7_1040300       198 GLKAGDIHGMKIVIEGLKALK-VDTLKSGIFNSFVQNSHYTEVTGLAIAIDTEMNEVCSATYIGIHPICV
                        111111111111111111111 1111111111111112222222222222222222222  2222  222
PF3D7_1254800       164 MNYETINAFIAKTIEELEGIPGITKLFGAKISQFVTPAVFRKPMSLVETILSEKKKLCL--CAANKNELL


PF3D7_1040300       267 VREKLGVIPKAGGTMVKQKDAITNVLKQALEKATQSAEA
                          2    222  22222222222222222222222  22
PF3D7_1254800       232 CRG----MNP--NVPETLPKKIEVAVNEVLSSVND--TW
```

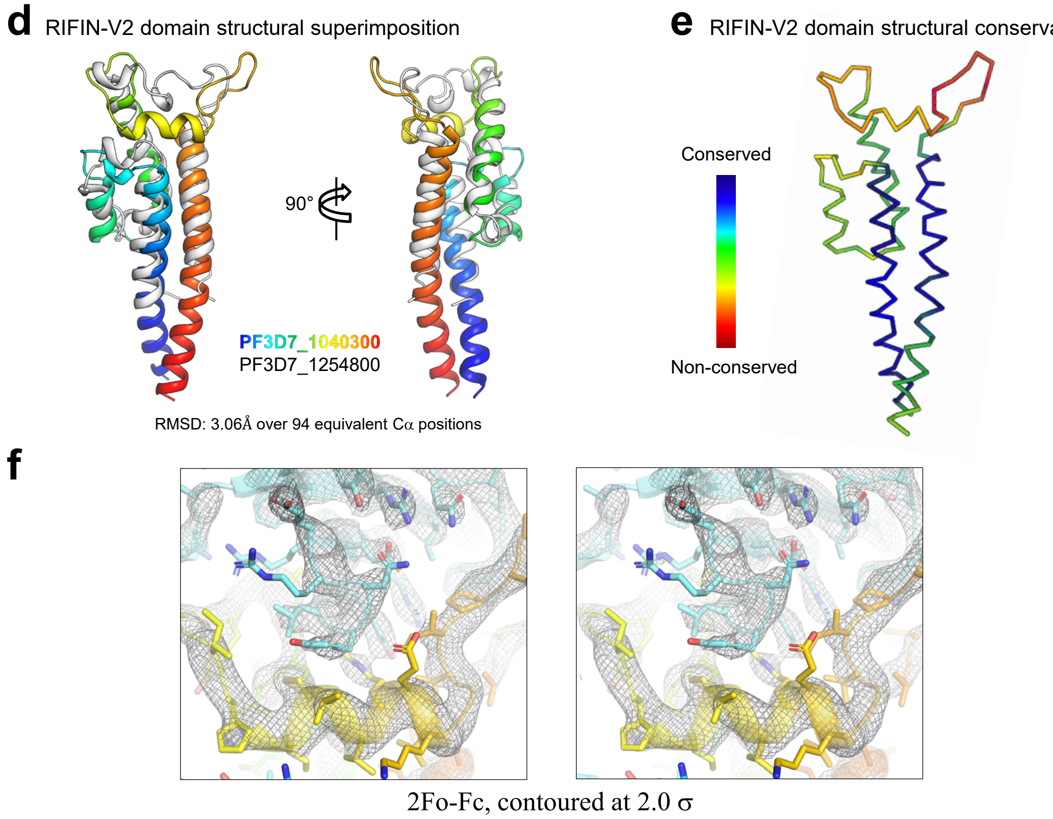

Supplementary Fig. 5 RIFIN-V2 structure and conservation. a, Ribbon representation of RIFIN-V2 (PF3D7_1040300, RIF1) structure viewed from two perpendicular angles. **b**, Surface sequence conservation analysis on PF3D7 RIFIN-V2 domain. **c**, Pairwise structural alignment between V2 domain of two RIFINs (PF3D7_1040300 and PF3D7_125480) by FATCAT server. **d**, Structural comparison between V2 domain of two RIFINs (PF3D7_1040300 in rainbow and PF3D7_125480 in white) in orthogonal views. **e**, Structural conservation of RIFIN-V2 domain as assessed by DALI server. **f**, Stereo image of the electron density map (2Fo-Fc, contoured at 2.0 σ) and the structure of the LAIR1-RIFIN binding interface.

a Co-IP assay by protein-A showing LAIR1-binding by three non-3D7 RIFINs

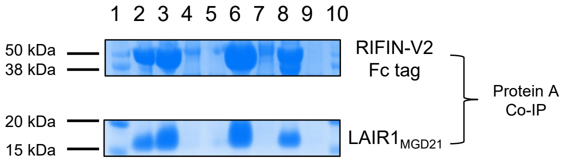

1. Marker
2. PF3D7_1040300
3. ETW15147.1_FVO
4. ETW19990.1
5. ETW43941.1
6. KNG75491.1_IGH-CR14
7. KNG75836.1
8. KOB85271.1_Dd2
9. CDO61877.1
10. Marker

b

```
KNG75491.1_IGH-CR14     AAAAQEAIAKGTAAGKIAGDIHGMEIVIEGLK-VLEVHRLESGIFNSFVNNSHYTEVTKL
ETW19990.1              VTAKELAEKAGAAAGLKAGDALGMEIVIEGLK-VLEVHTLESGIFNSFVNNSHYTEVTKL
ETW15147.1_FVO          AAAMELAKQAGATAGLKAGNTHGMNIVIHYLK-GLKVDTLKSGIFNSFVNNSHYTEVTGL
PF3D7_1040300           VTAKELAEKAGAAAGLKAGDIHGMKIVIEGLK-ALKVDTLKSGIFNSFVNNSHYTEVTGL
ETW43941.1              VTAKKLAAEAGAAAGLKAGDALGMKIVIKGLK-ALKVDTLKSGIYKTFVTTKRYSEVTGL
CDO61877.1              AAAKKAAIAKGLLFGKIVGDAKGMEIVIKGLD-VLKADKLIPEIFNSFKNSRHYTEVLDL
KOB85271.1_Dd2          AAATELAKQAGAAAGLKAGDAAGLNVVIGSLKRYLYIDKLNETPLQSFFTSTSYNDVTAI
KNG75836.1              VAAAEAAMAEGLAEGIKAGQVAGLAKFIDGFKDVFIMDSLNGTQLKSLFTLQNYTNFPNL
                        .:* : *    *    *   .*:  *:   .*   :.   :   . *      ::: .    *.:.   :

KNG75491.1_IGH-CR14     AIAIDTEMNEVCSATSIG--SQPICALRGKLGLVAKPGQSKVEQIDAITKVLKQALEKAT
ETW19990.1              ASVIDTEMNKVCFG------SHPICALREKLGLVAVPGRKMVNQKDAITKVLKQALEKAT
ETW15147.1_FVO          VDIINTQMNEVCSATYIG--THPICVVRNALGLEAVPGKAKVEQIDVIRRVLNEALEKAT
PF3D7_1040300           AIAIDTEMNEVCSATYIG--IHPICVVREKLGVIPKAGGTMVKQKDAITNVLKQALEKAT
ETW43941.1              VDIIDTEMNKVCSVTYVG--DQSICAVRNALGVIPKPGGTMVNQKDAITNVVTDFVDKAK
CDO61877.1              VDIINTQLNTTCSTGATAHVQNSICVVRETLGVIPKAGGKMVEQKTAITNVLDKALAKAT
KOB85271.1_Dd2          ANAIDAEMDASCGLSSFG--THTICALRGKLGLEAVPGGTMVNQKDAITRMINGLVGKAK
KNG75836.1              TRLINEELNKACSVWLTG--SNPICEVRTKLGLVAQAGQKWVEPESIIKLHVDKIVAESQ
                        .   *: :::   *          :.** :*   **: .  .*    *:      *    :    : ::

KNG75491.1_IGH-CR14     SSANAKATQVAATKTAALETAQKGAIEA
ETW19990.1              SSANAKATQVAATKTAALETAQKGAIEA
ETW15147.1_FVO          SSANAKATQVAADKTAEFKIANVGAVES
PF3D7_1040300           QSAEALSETTAEDVAAKLTAQKTGAINT
ETW43941.1              LSADTEAANVIKATSSKIIAKQTTAINS
CDO61877.1              SSANAAAKIAKKEVTAAAIEKNTGAVGT
KOB85271.1_Dd2          GTAKAVADSKNAEVVAELTAQKTDAINT
KNG75836.1              GAAAGVTKTVTEEATTTLTAQKTGVVQT
                         :*   :          :      :   .: :
```

Supplementary Fig. 6 Identification of additional LAIR1 binding RIFINs from non-3D7 strains. a, Co-immunoprecipitation showing LAIR1-binding by three non-3D7 RIFINs. The SDS-PAGE runs were repeated at least twice. **b,** RIFIN V2 sequence alignment (RIFINs shown by co-immunoprecipitation to be positive for LAIR1 binding labeled in red font).

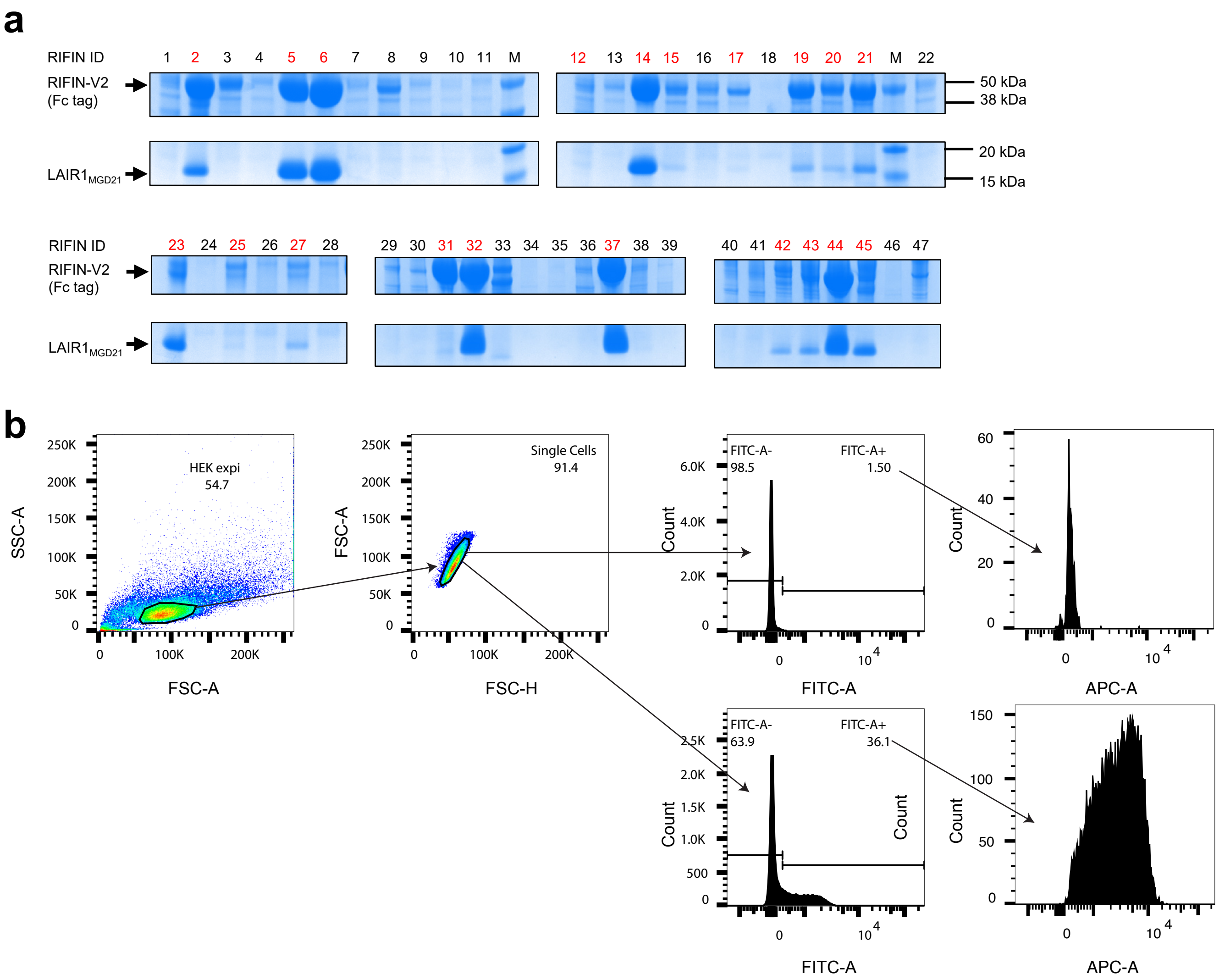

Supplementary Fig. 7 Characterization of RIFIN-LAIR1/LILRB1 interaction. a, Coomassie blue-stained SDS-PAGE to assess the co-IP between the LAIR1 domain of MGD21 and various RIFINs. (Related to Fig. 4c with additional Fc-tagged RIFIN-V2 bands shown) Positive LAIR1 binding RIFIN IDs are indicated in red. Lanes marked with "M" are molecular ladders with molecular weights as marked. The SDS-PAGE runs were repeated at least twice. **b**, Gating strategy to determine the percentage of RIFIN positive HEK cells (FITC-A + compartment) and RIFINs that binds to LAIR1 antibodies (APC-A compartment).
