# Supplementary material for: Structural basis of LAIR1 targeting by polymorphic Plasmodium RIFINs
Source: Nat Commun. 2021 Jul 9;12:4226. doi: 10.1038/s41467-021-24291-6 (PMC8270905; doi:10.1038/s41467-021-24291-6)
Supplement: Supplementary file 2 — Description of Additional Supplementary Files [file 41467_2021_24291_MOESM2_ESM.pdf]

Description of Additional Supplementary Files

File Name: Supplementary Data 1
Description: List of three-residue-signature matched RIFINs and their amino-acid sequences.
