# Supplementary material for: Structural basis of LAIR1 targeting by polymorphic Plasmodium RIFINs
Source: Nat Commun. 2021 Jul 9;12:4226. doi: 10.1038/s41467-021-24291-6 (PMC8270905; doi:10.1038/s41467-021-24291-6)
Supplement: Supplementary file 4 — Reporting Summary [file 41467_2021_24291_MOESM4_ESM.pdf]

nature research

Corresponding author(s): Peter D. Kwong, Kai Xu

Last updated by author(s): 05/22/2021

Reporting Summary

Nature Research wishes to improve the reproducibility of the work that we publish. This form provides structure for consistency and transparency in reporting. For further information on Nature Research policies, see our Editorial Policies and the Editorial Policy Checklist.

Statistics

For all statistical analyses, confirm that the following items are present in the figure legend, table legend, main text, or Methods section.

n/a	Confirmed	
☐	☒	The exact sample size (n) for each experimental group/condition, given as a discrete number and unit of measurement
☒	☐	A statement on whether measurements were taken from distinct samples or whether the same sample was measured repeatedly
☒	☐	The statistical test(s) used AND whether they are one- or two-sided<br>*Only common tests should be described solely by name; describe more complex techniques in the Methods section.*
☒	☐	A description of all covariates tested
☒	☐	A description of any assumptions or corrections, such as tests of normality and adjustment for multiple comparisons
☐	☒	A full description of the statistical parameters including central tendency (e.g. means) or other basic estimates (e.g. regression coefficient) AND variation (e.g. standard deviation) or associated estimates of uncertainty (e.g. confidence intervals)
☒	☐	For null hypothesis testing, the test statistic (e.g. F, t, r) with confidence intervals, effect sizes, degrees of freedom and P value noted<br>*Give P values as exact values whenever suitable.*
☒	☐	For Bayesian analysis, information on the choice of priors and Markov chain Monte Carlo settings
☒	☐	For hierarchical and complex designs, identification of the appropriate level for tests and full reporting of outcomes
☒	☐	Estimates of effect sizes (e.g. Cohen's d, Pearson's r), indicating how they were calculated

Our web collection on statistics for biologists contains articles on many of the points above.

Software and code

Policy information about availability of computer code

Data collection	X-ray crystallographic data were collected using SERGUI provided by SERCAT at Advance Photon Source. BD FACSDIVA (v6.2) were used for flow cytometry data collection. Electron microscopy images were collected using SerialEM (v3.8) or EPU (v2).
Data analysis	HKL2000 was used for crystal diffraction image processing. Phaser (v1.3) in Phenix (v1.18.2) was used for crystal structure solution. The structural models of antibody variable region were generated with PIGSpro. Coot (v0.9) was used for model building. Phenix was used for crystallographic refinement. Molprobity (v4.2) was used to check geometry. PyMOL (v1.8.6) were used to generate crystal structure figures. Electron microscopy particles were picked using e2boxer from the EMAN2 (v2.91) software package. Reference-free 2D classification was performed using EMAN2 (v2.91) and SPIDER (v25.00). 3D reconstruction was performed using SPIDER (v25.00) , cryoSPARC (v2.15) and FREALIGN (v9), with initial 3D references generated with EMAN2 (v2.91). Flow-Jo (v10.1) was used for all FACS analyses.

For manuscripts utilizing custom algorithms or software that are central to the research but not yet described in published literature, software must be made available to editors and reviewers. We strongly encourage code deposition in a community repository (e.g. GitHub). See the Nature Research guidelines for submitting code & software for further information.

Data

Policy information about availability of data

All manuscripts must include a data availability statement. This statement should provide the following information, where applicable:

- Accession codes, unique identifiers, or web links for publicly available datasets
- A list of figures that have associated raw data
- A description of any restrictions on data availability

Coordinates for the crystal structures determined in this study have been deposited in the Protein Data Bank as PDB 7JZ1, 7JZ4, 7JZI and 7JZK. PDB IDs 3KGR and 6ZDX were downloaded from Protein Data Bank. These data were analyzed in Figs. 1, 2, 3 and Supplementary Figs. 1, 5. Raw data (SDS-PAGE gel image) associated

with Figs 2c, 3b, 3e, 4c, S6 and S7a are provided in Source data file. Plasmodium genome database can be accessed on the web (plasmodb.org).

Field-specific reporting

Please select the one below that is the best fit for your research. If you are not sure, read the appropriate sections before making your selection.

[x] Life sciences [ ] Behavioural & social sciences [ ] Ecological, evolutionary & environmental sciences

For a reference copy of the document with all sections, see nature.com/documents/nr-reporting-summary-flat.pdf

Life sciences study design

All studies must disclose on these points even when the disclosure is negative.

Sample size	Number of images for X-ray crystallographic data collection were determined by timer availability, crystal quality, and data needed to achieve a reliable interpretation of the structural data.
Data exclusions	All data were processed and analyzed.
Replication	BLI experiments and SDS-PAGE image quantification were repeated three times. All attempts at replication were successful. No additional attempts of replication were made other than mentioned here.
Randomization	No randomization was used in this study. The experimental procedure for structural determination and binding test has been well documented and routinely followed. It is independent of randomization.
Blinding	No blinding was used in this study. The experimental procedure for structural determination and binding test has been well documented and routinely followed. It is independent of individual variation.

Reporting for specific materials, systems and methods

We require information from authors about some types of materials, experimental systems and methods used in many studies. Here, indicate whether each material, system or method listed is relevant to your study. If you are not sure if a list item applies to your research, read the appropriate section before selecting a response.

Materials & experimental systems

n/a	Involved in the study	
[ ]	[x]	Antibodies
[ ]	[x]	Eukaryotic cell lines
[x]	[ ]	Palaeontology and archaeology
[x]	[ ]	Animals and other organisms
[x]	[ ]	Human research participants
[x]	[ ]	Clinical data
[x]	[ ]	Dual use research of concern

Methods

n/a	Involved in the study	
[x]	[ ]	ChIP-seq
[ ]	[x]	Flow cytometry
[x]	[ ]	MRI-based neuroimaging

Antibodies

Antibodies used	MGD21, MGD21-UCA, MGC34, MGB47, MGJ5, MDA1, MDB1, MDC1 and BKC3 were made in lab. Alexa Fluor 647-conjugated goat anti-human IgG from Jackson ImmunoResearch, cat. no. 109-606-170, 1.5mg/ml stock, dil 1/500. Rabbit anti-HA tag, from Sigma, cat. no. H6908, 5 µg/ml. Alexa Fluor 488-conjugated goat anti-rabbit IgG, from Life Technologies, cat. no. A11034, lot no. 1670152, 5 µg/ml.
Validation	The antibody produced in lab were confirmed by DNA sequencing, gel-filtration and SBS-PAGE and Co-IP binding analysis. Representative result of LAIR1-containing antibodies were shown in Fig S1. Characterization of LILRB1-containing antibodies was published in Chen, Xu et al, Nature 2021. The species and application of all commercial antibodies were described above and in manuscript. Commercial antibody specificity was based on antigen-binding assay or ELISA. Detail were provided on product webpage.

Eukaryotic cell lines

Policy information about cell lines

Cell line source(s)	Expi293F and Expi293_Gnti- cells were purchased from Thermo Fisher Scientific for protein production.
Authentication	The cells were used directly from the commercial sources following manufacturer suggestions, without further authentication.

Mycoplasma contamination	All the cell lines tested negative for Mycoplasma contamination.
Commonly misidentified lines (See ICLAC register)	No commonly misidentified cell lines were used in the study.

Flow Cytometry

Plots

Confirm that:

- [x] The axis labels state the marker and fluorochrome used (e.g. CD4-FITC).
- [x] The axis scales are clearly visible. Include numbers along axes only for bottom left plot of group (a 'group' is an analysis of identical markers).
- [x] All plots are contour plots with outliers or pseudocolor plots.
- [x] A numerical value for number of cells or percentage (with statistics) is provided.

Methodology

Sample preparation	Expi293F cells purchased from Thermo Fisher Scientific were cultured at 0.7mi cells/ml one day before transfection with RIFIN candidates. Transfectants were collected 72hours post transfection using centrifugation. Cells were stained with antibodies for the detection of RIFIN and HA tag.
Instrument	BD FACSCanto I Cat no. 337175
Software	FACS Diva (version 6.2) was used for acquisition of samples. Flow-Jo (version 10.1) was used for all the FACS analyses.
Cell population abundance	Under the histogram panel, The RIFIN-HA-488 positive population was determined by setting a fraction excluding all non stained cell controls.
Gating strategy	Gating in Figure 3d followed the standard gating procedures. Under the pseudocolor plot, live cells were in grouped in clear population in the FSC/SSC plot. The Singlets were gated by eliminating the shades of the edge. RIFIN-HA-488 positive population was determined by comparing to the unstained cells. IgG-A647 was visualized using histogram.

- [x] Tick this box to confirm that a figure exemplifying the gating strategy is provided in the Supplementary Information.
